# 3D Magnetic Resonance Spirometry

**DOI:** 10.1038/s41598-020-66202-7

**Published:** 2020-06-15

**Authors:** Tanguy Boucneau, Brice Fernandez, Peder Larson, Luc Darrasse, Xavier Maître

**Affiliations:** 1 Université Paris-Saclay, CEA, CNRS, Inserm, BioMaps, Orsay, France; 2 Applications & Workflow, GE Healthcare, Orsay, France; 3 Department of Radiology and Biomedical Imaging, University of California San Francisco, San Francisco, CA USA

**Keywords:** Biomedical engineering, Respiratory tract diseases, Imaging techniques

## Abstract

Spirometry is today the gold standard technique for assessing pulmonary ventilatory function in humans. From the shape of a flow-volume loop measured while the patient is performing forced respiratory cycles, the Forced Vital Capacity (FVC) and the Forced Expiratory Volume in one second (FEV_1_) can be inferred, and the pulmonologist is able to detect and characterize common respiratory afflictions. This technique is non-invasive, simple, widely available, robust, repeatable and reproducible. Yet, its outcomes rely on the patient’s cooperation and provide only global information over the lung. With 3D Magnetic Resonance (MR) Spirometry, local ventilation can be assessed by MRI anywhere in the lung while the patient is freely breathing. The larger dimensionality of 3D MR Spirometry advantageously allows the extraction of original metrics that characterize the anisotropic and hysteretic regional mechanical behavior of the lung. Here, we demonstrated the potential of this technique on a healthy human volunteer breathing along different respiratory patterns during the MR acquisition. These new results are discussed with lung physiology and recent pulmonary CT data. As respiratory mechanics inherently support lung ventilation, 3D MR Spirometry may open a new way to non-invasively explore lung function while providing improved diagnosis of localized pulmonary diseases.

## Introduction

Gas ventilation, together with blood perfusion and septal gas transfer, ensures the global lung function in humans. During breathing, ventilation is strongly related to lung mechanical behavior as it is characterized by the inflation and deflation of pulmonary alveoli while gas flows and diffuses in and out the lung parenchyma. Ventilation quality and respiratory diseases are clinically investigated with spirometry as a part of routine pulmonary function tests^1^. However, the outcome of spirometry is global as it integrates the function of the whole organ at the subject’s mouth. In spite of intrinsic limitations which have hindered its application in the lung, Magnetic Resonance Imaging (MRI) have been investigated for the assessment of lung ventilatory function at the regional scale. Thus, MRI may provide three-dimensional images of human lungs in motion without ionization hazard to map lung ventilation either:

1. through the direct or indirect gas-based quantification of local gas volumes in the lung parenchyma, or
2. through the direct tissue-based assessment of respiratory mechanics.

In the first approach, it can be directly performed by imaging an inhaled tracer gas like hyperpolarized ^3^He^2^ or ^129^Xe^3^, or fluorinated SF_6_ or CF_4_^4^ with dedicated hardware. It is also indirectly performed by administering a gas contrast agent, like oxygen, to enhance the parenchymal signal along the respiration in ^1^H MRI^5^. Hyperpolarized ^129^Xe MRI even makes possible the exploration of the three aspects of the lung function: ventilation, perfusion and gas transfer. Nevertheless, these different strategies require additional hardware (e.g. polarizer, transmit and receive RF channels) and often rely on specific gas administration protocols, which altogether have limited their application to the clinic.

In the second approach, lung ventilation is inferred from lung volumetric changes in standard dynamic ^1^H MR imaging. Basic methods study the time evolution of lung volumes^6,7^, the frequency evolution of the MR signal^8^, or the behavior of respiratory muscles (diaphragm^9^ and intercostal muscles in the chest wall^10^ mainly). Some of these techniques, like Fourier decomposition MRI^8^ and PREFUL MRI^11,12^, also give access to blood perfusion information. However, those techniques are usually limited to 2D imaging and they eventually probe the lung as a whole.

The feasibility of 3D regional ventilation was explored with proton MRI following two main strategies. The first one consisted in tagging a 2D grid inside the thorax with spatially selective spin saturation techniques^13–15^. By interleaving the application of this tagging pulse with the rapid acquisition of 2D MR images, and by analyzing the regional deformation of this grid along the free-breathing respiratory motion of the subject as a post-processing step, it was possible to deduce the local deformation of the lung, calculate regional volume changes, represent regional spirometry-like flow-volume loops and estimate mechanical strain parameters. The second strategy was very similar to the first one and aimed at estimating regional ventilation parameters by non-elastic registration of dynamic MR images. However, it relied on the fiducial landmarks of the physical boundaries of the organ, which included the pleural space and the main vascular system, instead of the deformation of the added tagged grid^16,17^.

The latter strategy presents two main advantages. First, it can be easily implemented on almost any MRI system as it is based on ^1^H MRI and standard dynamic sequences. Acquisition protocols featuring spin-tagging are usually more complex in comparison. Second, it measures lung ventilation only on a mechanical behavior basis. It means that the only required information is the local displacement of each piece of lung tissue, which can be directly addressed with dynamic lung imaging and several standard post-processing steps.

However, only one image slice is usually considered because of the scan time constraints imposed by 2D multi-slice or 3D MR imaging. It is still challenging today to obtain 3D images with millimeter spatial and sub-second time resolutions to effectively probe the respiratory dynamics in freely breathing human subjects^18^.

In this work, we developed a global strategy and framework to jointly analyze the ventilatory function and the mechanical behavior of the lung. We implemented a 3D Ultrashort Echo Time (UTE) sequence with 1.5 mm isotropic voxel size and 2 ms repetition time (TR) at 3 T to acquire 3D images for 32 phases over a time-averaged human respiratory cycle by retrospective self-gating. This pulse sequence was demonstrated to be particularly well adapted to dynamic lung imaging^19^. Once displacement fields were computed from the 32 registered images, we prospected a set of metrics to obtain time-resolved 3D voxel-wise information of pulmonary ventilation and mechanical behavior throughout the entire respiratory cycle. The overall methodology developed here was subsumed under the name 3D Magnetic Resonance Spirometry (or 3D MR Spirometry).

## Results

We tested 3D MR Spirometry on a healthy human volunteer (male, 26 years old). Three acquisitions were performed during the same imaging session and the volunteer was asked to perform three different types of breathing patterns as he laid supine in the MRI system:

- A normal breathing pattern: The volunteer was asked to breathe as spontaneously as possible.
- A thoracic breathing pattern: The volunteer was asked to breathe mostly with the intercostal muscles and without using the diaphragm.
- A diaphragmatic breathing: The volunteer was asked to breathe mostly with the diaphragm and without using the intercostal muscles.

**Figure 1:**
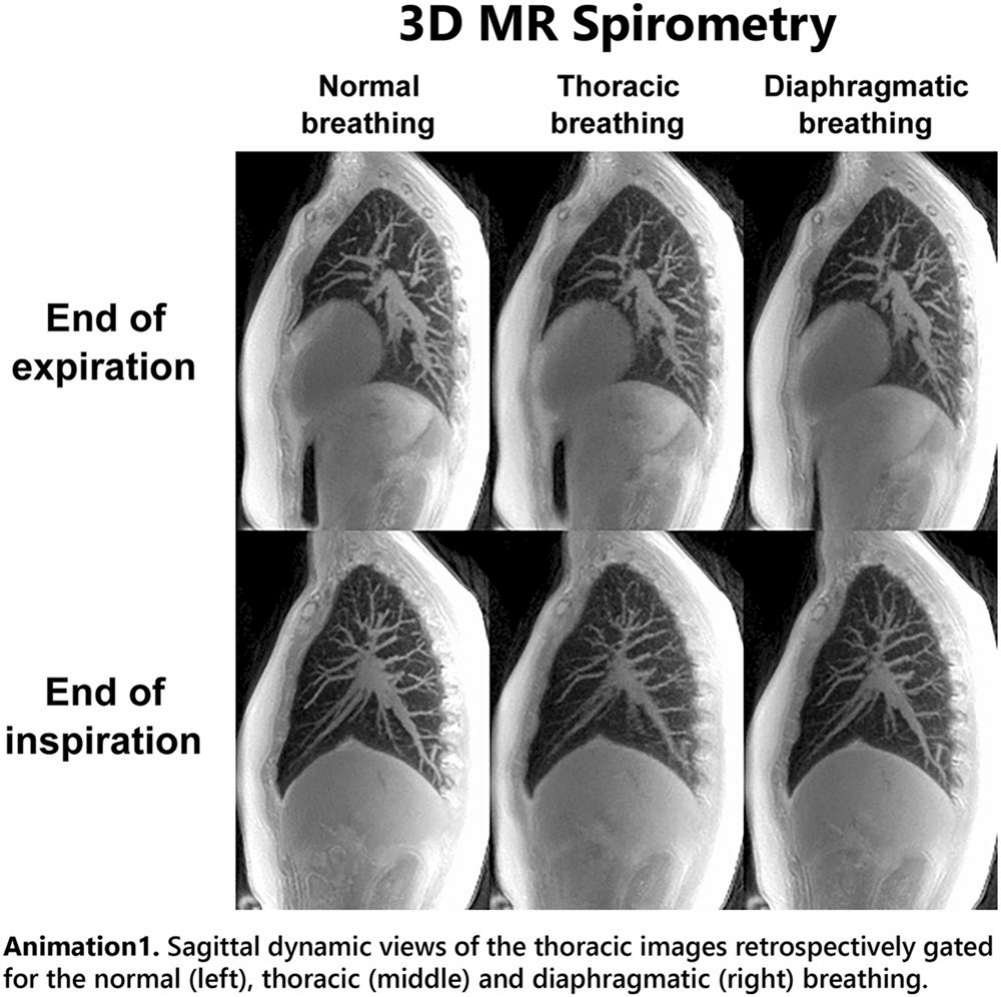
Sagittal views of the thoracic images retrospectively gated at the end of expiration (top) and at the end of inspiration (bottom) for the normal (left), thoracic (middle) and diaphragmatic (right) breathing patterns. On every image at the end of inspiration, the red curve depicts the border of the lung observed in the corresponding expiratory state shown above. As pointed out by the red arrows, it clearly stresses the respective motions of the diaphragm and the thoracic wall for each breathing pattern. The respiratory dynamics are provided as supplementary materials.

The motion corrected images obtained at the end of inspiration and expiration are presented in Fig. 1. They represent sagittal views of 3D images taken out of the 32 key images obtained along the time-averaged respiratory cycle of 3–5 s duration. With an approximate pseudo temporal resolution of 100–150 ms, we were able to study the respiratory mechanics.

### Hysteretic behavior of the deformation field inside the lung

**Figure 2:**
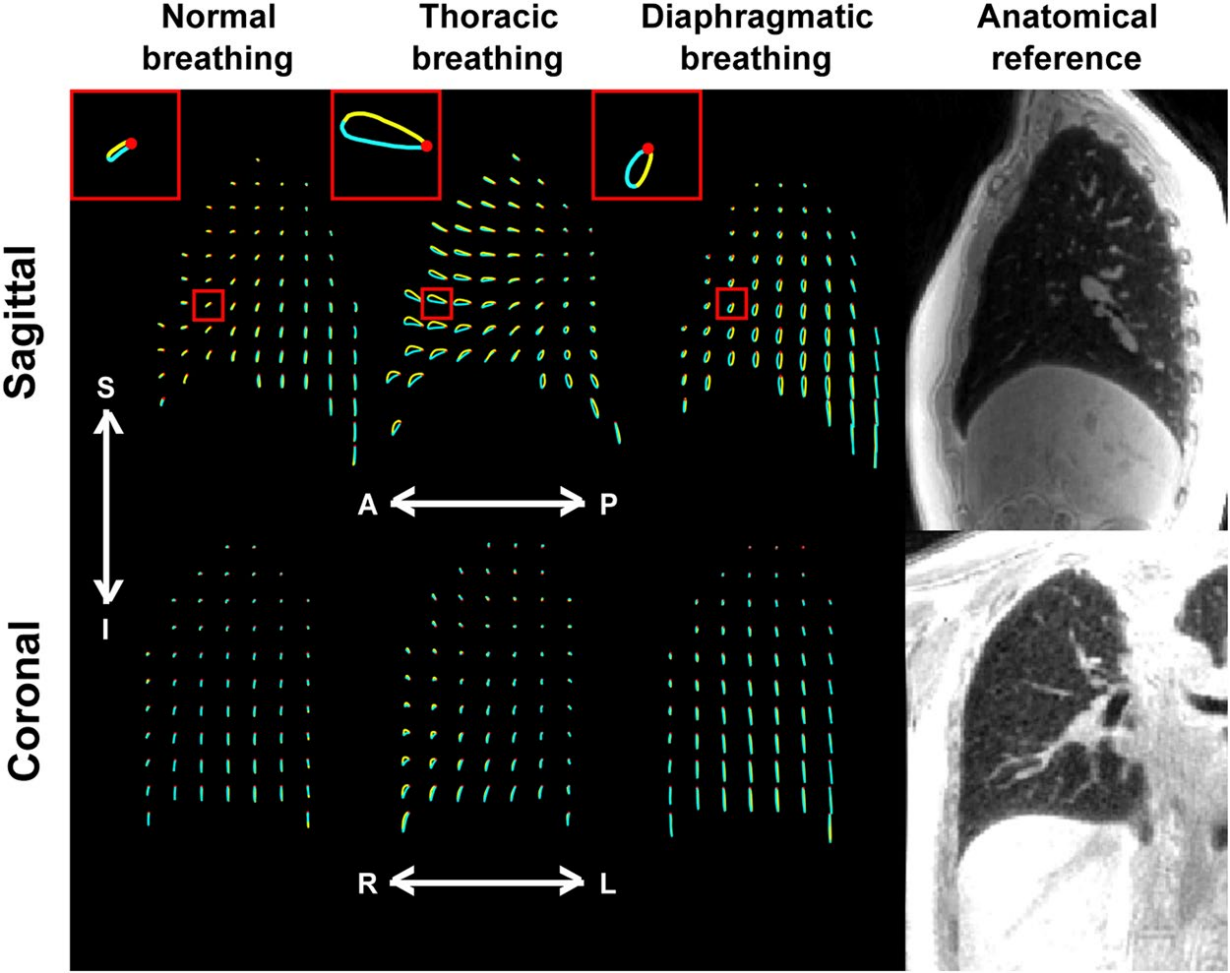
Sagittal (top) and coronal (bottom) views of the trajectories followed by elementary volumes of the right lung parenchyma, located at red dots positions at the end of expiration. For the sake of clarity, only one percent of the trajectories is shown. These trajectories are pictured for the normal (left), thoracic (middle) and diaphragmatic (right) breathing patterns. In each trajectory, the inspiration phase is represented in yellow and the expiration phase is represented in blue. In the top left-hand corner of each represented breathing pattern, a focus on one representative trajectory (location depicted by the small red squares) is given. A UTE MR image is given on the right of each row as an anatomical reference.

After elastic registration of the above-mentioned images to the image obtained at the end of expiration, used as reference, we can compute the trajectory followed by any elementary piece of lung parenchyma during the breathing cycle. The trajectories in the sagittal and coronal planes are illustrated in Fig. 2. For the sake of clarity, only one percent of the trajectories is shown. We observe that these trajectories describe closed loops everywhere in the lung, which locally sustain the motion hysteresis of any global respiration. They are primarily 2D trajectories in the sagittal plane for the three types of breathing. However, the shape and the amount of motion hysteresis varied a lot throughout the lung and between the different types of breathing. In normal breathing, there is very little motion hysteresis and the trajectories show an elongated shape oriented diagonally in the sagittal plane, with a principal direction of motion both along the superior-inferior and the anterior-posterior directions. For thoracic and diaphragmatic breathings, these trajectories are mainly oriented respectively along the anterior-posterior direction and along the superior-inferior direction, and the amount of motion hysteresis varies a lot throughout the organ.

Overall, for the three types of breathing patterns, motion amplitude and hysteresis are larger in the inferior than in the superior regions of the lung. In the case of thoracic breathing, they are larger in the anterior than in the posterior regions of the lung. Following the trends depicted by the close-ups on the top of Fig. 2, in normal breathing, the inspiratory motion goes downward and frontward – the lung preferentially expands towards the anterior and the inferior of the body – whereas, in thoracic breathing, it goes upward and frontward. In both normal and thoracic breathings, the respiratory motion remains counterclockwise. On the contrary, in diaphragmatic breathing, the respiratory motion goes clockwise, as if the tissues were first moving backward at the initiation of the inspiration. In a radically different behavior, they essentially move downward before going back upward with an expiratory frontward motion.

### Non-uniform regional volume expansions in the lung

**Figure 3:**
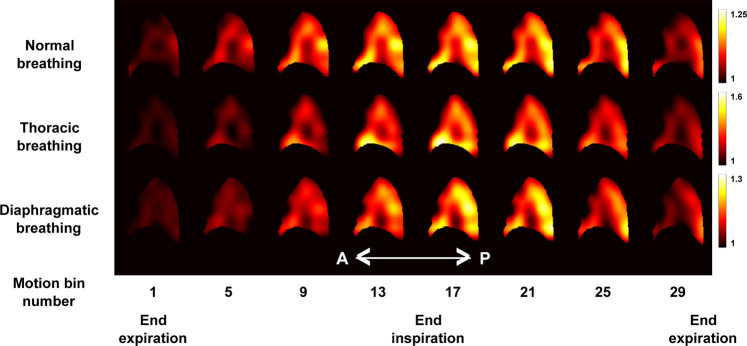
Representation of 8 out of 32 sagittal views (every 4^th^) showing the evolution of the $J$ map throughout the gated time-average respiratory cycle for the normal (top), thoracic (middle) and diaphragmatic (bottom) breathing patterns.

Using the Jacobian of the displacement field, $J$, with respect to the reference lung state at the end of the expiration at each voxel position and at each time-point of the time-average respiratory cycle, we computed the regional volume expansion. Figure 3 shows the $J$ maps obtained for the three types of breathing patterns at eight different phases of the respiratory cycle. We observe that the estimated values of $J$ globally vary a lot between breathing patterns because of the difference in breathing amplitudes discussed above. However, for each breathing pattern and everywhere in the lung, we observe that $J$ values monotonically increase during the inspiration phase and monotonically decrease during the expiration phase, which is expected. Moreover, for each breathing pattern and for each time-stamp, we observe that $J$ is not uniformly distributed within the organ. In all breathing types, we observe higher $J$ values in the inferior than in the superior regions of the lung, and larger values are reached in the posterior region of the lung. Furthermore, as we could expect, we observe a slight volume enhancement in the anterior region of the lung for thoracic breathing in comparison to normal and diaphragmatic breathings.

### Anisotropic deformation of the lung parenchyma

**Figure 4:**
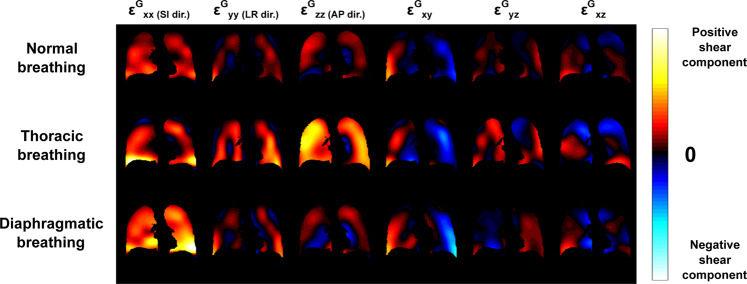
Coronal views of the different components of the Green-Lagrange strain tensor ${\varepsilon }_{xx}^{G}$ (along the superior-inferior direction), ${\varepsilon }_{yy}^{G}$ (along the left-right direction), ${\varepsilon }_{zz}^{G}$ (along the anterior-posterior direction), ${\varepsilon }_{xy}^{G}$, ${\varepsilon }_{yz}^{G}$, and ${\varepsilon }_{xz}^{G}$ for normal, thoracic and diaphragmatic breathing patterns.

In the Green-Lagrange strain tensor, ${\varepsilon }^{G}$, at each voxel and time position, the diagonal terms (${\varepsilon }_{ii}^{G}$) represent what could be called ‘contraction/compression’ or ‘expansion/stretching’ along each $i$ direction. The non-diagonal terms (${\varepsilon }_{ij}^{G}$, with $i\ne j$) represent what could be called ‘shear deformation’. Due to the symmetry of ${\varepsilon }^{G}$, we only calculated six different strain maps (${\varepsilon }_{xx}^{G}$, ${\varepsilon }_{yy}^{G}$, ${\varepsilon }_{zz}^{G}$, ${\varepsilon }_{xy}^{G}$, ${\varepsilon }_{yz}^{G}$, and ${\varepsilon }_{xz}^{G}$) at any phase of the respiratory cycle. They are represented in Fig. 4 for the inflation state obtained at the end of inspiration and for the three breathing patterns.

In Fig. 4, the maps of the diagonal terms of ${\varepsilon }^{G}$ (${\varepsilon }_{xx}^{G}$, ${\varepsilon }_{yy}^{G}$, and ${\varepsilon }_{zz}^{G}$) show that the strains along, respectively, superior-inferior, left-right, and anterior-posterior directions do not contribute to the same extent to the local lung ventilation. Therefore, ventilation is locally and globally anisotropic in the lung. Moreover, these strain contributions along the three main directions of space differ for the three types of breathing patterns. For normal breathing, the main strain components essentially occur along the superior-inferior (${\varepsilon }_{xx}^{G}$) and anterior-posterior axes (${\varepsilon }_{zz}^{G}$). However, for thoracic breathing, the main contribution is essentially along the anterior-posterior axis (${\varepsilon }_{zz}^{G}$), whereas it is along the superior-inferior (${\varepsilon }_{xx}^{G}$) axis for diaphragmatic breathing. For the three types of breathing, the strain contribution along the left-right direction (${\varepsilon }_{yy}^{G}$) is always the weakest. The visual analysis of the non-diagonal Green-Lagrange strain tensor components (${\varepsilon }_{xy}^{G}$, ${\varepsilon }_{yz}^{G}$, and ${\varepsilon }_{xz}^{G}$) is more difficult. However, for the three types of breathing, we still observe an anti-symmetry between the two lungs for the strain components within the coronal plane (${\varepsilon }_{xy}^{G}$) and within the axial plane (${\varepsilon }_{yz}^{G}$), but a symmetry within the sagittal plane (${\varepsilon }_{xz}^{G}$), which is what can be expected from a simplified model considering the two lungs behaving symmetrically.

**Figure 5:**
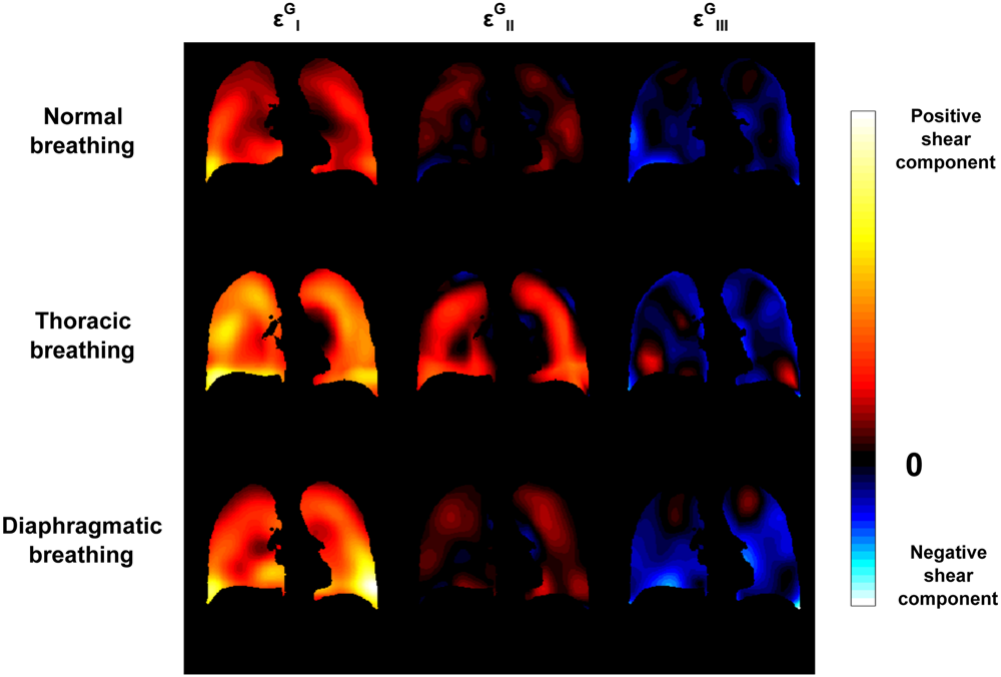
Coronal views of the three principal strain components, ${\varepsilon }_{I}^{G}$, ${\varepsilon }_{II}^{G}$, and ${\varepsilon }_{III}^{G}$, for normal, thoracic, and diaphragmatic breathing patterns.

The three main strains, ${\varepsilon }_{I}^{G}$, ${\varepsilon }_{II}^{G}$, and ${\varepsilon }_{III}^{G}$ with ${\varepsilon }_{I}^{G} > {\varepsilon }_{II}^{G} > {\varepsilon }_{III}^{G}$, extracted from the diagonalized Green-Lagrange strain tensor at each space and time point, condense the information contained in the six maps shown above in the three maps represented in Fig. 5. We observe again that the local lung deformation is anisotropic because, at each given location, ${\varepsilon }_{I}^{G}$, ${\varepsilon }_{II}^{G}$, and ${\varepsilon }_{III}^{G}$ show very different values. More precisely, we observe that tissue locally expands along two principal directions and contracts along the third one almost everywhere in the organ. For thoracic breathing, the secondary strain component is more strongly pronounced than for the normal and diaphragmatic ones.

**Figure 6:**
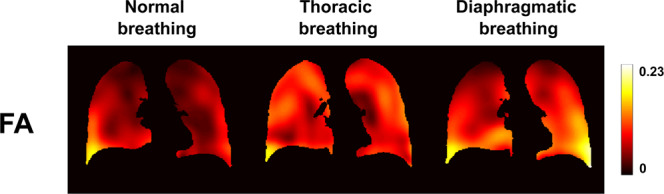
Coronal views of Fractional Anisotropy, $FA$, maps for normal, thoracic, and diaphragmatic breathing patterns.

The amount of strain anisotropy inside the lung parenchyma, quantified by the Fractional Anisotropy parameter, $FA$, is represented by the parametric maps of Fig. 6. Again, we observe differences between the three types of breathing on $FA$ maps. In particular, higher anisotropy is obtained in the lung base for normal and diaphragmatic breathings, whereas anisotropy is more uniform throughout the lung for thoracic breathing. Overall, the fractional anisotropy values remain low: $FA=(0.0640\pm 0.032)$ for normal breathing, $FA=(0.102\pm 0.035)$ for thoracic breathing, and $FA=(0.0953\pm 0.037)$ for diaphragmatic breathing (given as $({\rm{mean}}\pm {\rm{standard}}\,{\rm{deviation}})$ over the entire lung volume).

### 3D flow-volume loop maps

**Figure 7:**
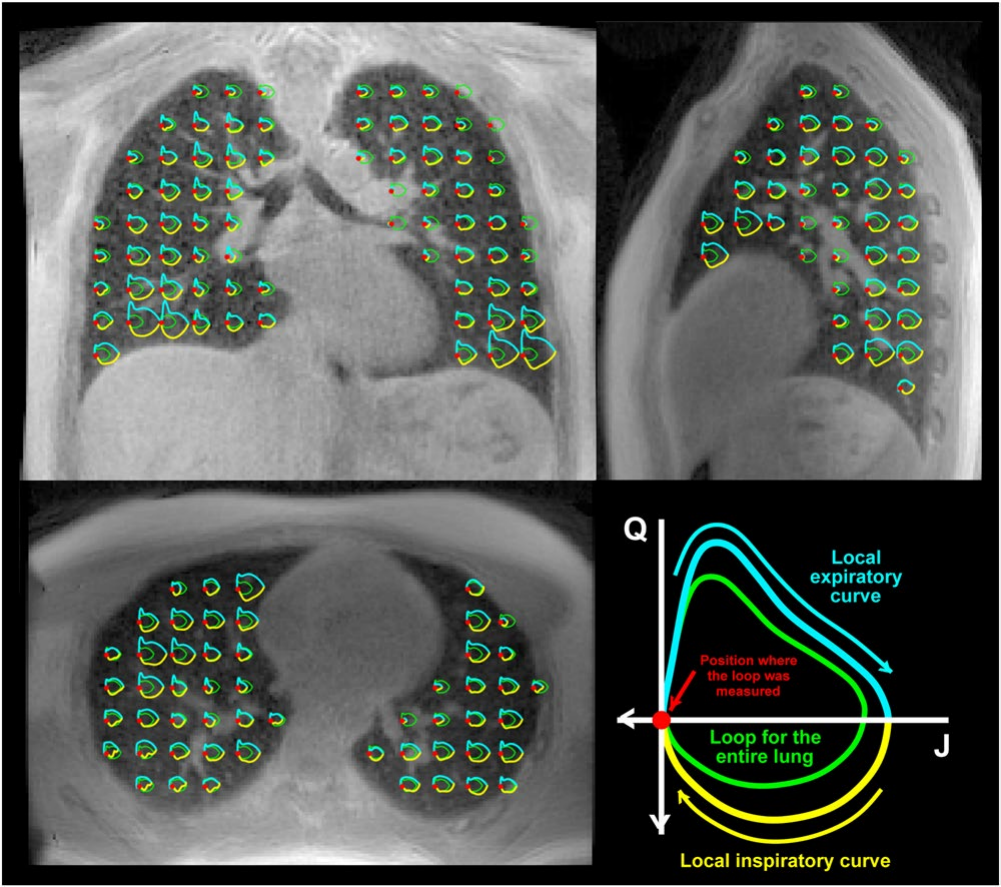
Axial, sagittal and coronal views from the 3D UTE dataset at the end of expiration, and over which are superimposed the regional flow-volume loops estimated with 3D MR Spirometry (only one every 20^th^) for thoracic breathing. For each local loop, the global loop representing the flow-volume for the entire lung is also drawn for reference (green). In the bottom right-hand corner, we showed the color and axis conventions for the local and global loops.

We introduce 3D MR Spirometry by maps of first time derivatives of the metrics presented above. In reference to standard spirometry, the time derivative of the Jacobian, $Q$, provides the instantaneous regional gas flow inside the lung parenchyma, while $J$ provides the instantaneous gas volume expansion with respect to the reference lung state at the end of expiration. Local flow-volume loops, $Q=f(J)$, are represented anywhere in the lung in Fig. 7 as $J$ and $Q$ are computed at any voxel position and at any phase of the time-averaged respiratory cycle.

Such an outcome is well known in the field of lung physiology as being the main information returned by standard spirometers for the entire organ. With our technique, the same measurement is performed at the regional scale, extracted directly from the 3D MR images. On Fig. 7, the representation of the flow-volume loops follows the conventions used in standard spirometry with the expiration phase on the top and the deflation state to the right. In this representation, some local loops in the lung look very similar to global loops usually observed for the whole lung in standard spirometry. By comparing the local loops (represented in yellow and blue, respectively for inspiratory and expiratory phases) and the one obtained for the total lung volume (represented in green), the local variability of the sizes and shapes of the loops are more easily revealed throughout the lung.

## Discussion

From the pulmonary deformation fields extracted out of a set of MR lung images, the trajectory that each lung elementary volume follows is determined, the tissue strain components are computed, the strain anisotropy is characterized, the regional volume change is quantified with respect to a given reference state and the rate of volume change, hence the local gas flow, is derived. Local flow-volume loops are produced to provide graphic analysis of the local ventilation occurring during a time-average respiratory cycle. In the same lines of standard spirometry, maps of time-averaged local tidal volumes (LTV) and time-averaged local expiratory volumes in the first second (LEV_1_) could be calculated instead of Forced Vital Capacity (FVC) and Forced Expiratory Volume in the first second (FEV_1_). The accuracy and the reproducibility of standard spirometry rely on the calibration and the maintenance of the measurement apparatus, the operating technique, and the effort the patient is able to provide in order to effectively and repeatedly achieve forced respirations. FVC and FEV_1_ result from coached forced exhalation maneuvers whereas LTV and LEV_1_ result from spontaneous free breathing. Thus, beyond the dimensionality and locality gains, 3D MR spirometry will inherently provide different information.

In spontaneous breathing, standard spirometry loses all sensitivity to ventilation defects and any disease specificity as the global flow-volume loops are generally affected only when the lung is challenged to the limit of its abilities. Even at the high and low pressures that are reached during coached forced breathing, FVC and FEV_1_ may fail revealing or staging lung dysfunction as it was brought to light early on by hyperpolarized gas MRI when the recorded percentage of ventilation defects did not always correlate with standard spirometry^20–23^. In 3D MR Spirometry, because of the long total scan time, only spontaneous breathing can be reasonably performed by the patient. However, the related loss of sensitivity and specificity may be compensated by the localization of the measurements. This would be particularly effective in the case of regional lung afflictions, for which the effect of the disease over the whole lung, as rendered at the mouth in standard spirometry, might be averaged out by the normal respiratory function shown everywhere else in the lung parenchyma. More generally, as lung diseases are spatially heterogeneous, the spatial resolution that 3D MR spirometry provides is needed to localize dysfunctional components from functional components within the lung. In 3D MR Spirometry, by mapping ventilation and strain behavior in the lung during free-breathing, similar or even higher sensitivity and specificity can be expected to localized lung defects and diseases like emphysema, lung cancer, radiation fibrosis, and generally any Chronic Obstructive Pulmonary Disease (COPD).

Our technique opens new ways to understand the origin of a given disease and to explore lung afflictions directly where they develop: Is it related to the diaphragm or the chest wall? Is it unilateral or bilateral? Furthermore, the measurement does not require any calibration procedure and it is not operator dependent. The measurement of the different parameters in the entire organ offers even improved normalization perspectives for intra-subject and inter-subject comparisons (by dividing a parameter by its average value over the entire organ, or by fitting the final parametric maps onto a representative atlas of the human lung, for example). Three-dimensional MR Spirometry is not just a 3D spirometry technique. It provides many new parameters to regionally describe the lung function and which might be highly valuable for lung physiopathology. Their contribution to the diagnosis of the pulmonary function remains to be elaborated.

Results obtained here for a healthy subject are instructive in terms of pulmonary mechanics, and this is well illustrated in the trajectories shown in Fig. 2. If we assume that normal breathing results from the concurrent action of the diaphragm and the intercostal muscles, the normal trajectories are expected to stand between the two extreme diaphragmatic and thoracic cases. In normal breathing, trajectories are indeed diagonally oriented and they show, as a result, very little amount of motion hysteresis. The latter could also be explained by the fact that total ventilation volumes were experimentally lower during the normal breathing experiment than during the thoracic and diaphragmatic ones.

The main strength of the technique is to be based on 3D motion corrected MR images, namely on complete 4D MR dataset. As seen above, the parameters we have explored can be quantified, first, everywhere in the lung, second upon the actual 3D motion field of the lung parenchyma. The latter is very important because, from a Lagrangian point of view, each elementary volume of lung tissue follows a trajectory in a 3D space. If this trajectory is extracted from a space with a lower number of dimensions (2D MR images or 2D projection images, for example), the information is inherently incomplete and leads to quantification biases. It was the case when the extracted deformation field was limited to the coronal plane such that any component of motion along the anterior-posterior axis was discarded^13,14^. Even though this work was further extended to ‘real-time’ MR spirometry with 7 time-stamps per respiratory period, it was still based on 2D coronal images and grid-tagging^15^. Thus, in either case, the anterior-posterior component of motion was not measured and its contribution could not be considered while processing the flow-volume loops and associated parameters, whereas it can be crucial as seen here in the case of thoracic breathing for example (Fig. 4). Other groups proposed multi-slice 2D acquisitions in the sagittal plane, which largely improved the estimation of motion fields and alleviated the biases of a reduced dimensionality, even though it still maintains a certain bias in the acquisition with respect to the 3D acquisitions performed here. These groups either used dynamic grid-tagging in ^3^He MRI^24^ or recorded static images at different levels of lung inflation for subsequent breath-holds^16,17^. In the first case, it was still based on grid-tagging and it carries the burden of hyperpolarized gas MRI. In the second case, as the images were acquired in static mode, every dynamic aspect of lung mechanics was lost, which is detrimental to the central notion of time in the phenomena involved in lung function as integrated in standard spirometry.

Compared to approaches with spin-tagging ^1^H MRI, 3D MR Spirometry has the advantage to rely on simpler 3D image acquisition processes with shorter total acquisition times and lower Specific Absorption Rate (SAR) values. The accuracy of 3D MR Spirometry primarily depends on the presence of local structural elements like fiducial landmarks and borders that show up in the images to promote viable registration of acquired dynamic MR images. Thus, the image registration process was expectedly more difficult in distal lung regions, where fewer landmarks are available, than in medial lung regions, which are highly perfused by clearly visible large blood vessels. Nevertheless, with the implemented UTE pulse sequence, even short ${T}_{2}^{\ast }$ tissues like the lung parenchyma could be imaged and delineated, such that registration was successfully performed everywhere in the lung, and flow-volume loops and correlated parameters were deduced in any given lung region.

To our knowledge, the most advanced work in the field was carried by Kolb *et al*. in 2016^25^. They also made use of retrospectively self-gated 3D MRI in free-breathing subjects. However, instead of separately segmenting and registering the images without any prior, they undertook joint segmentation and registration based on a hypothetical 3D lung model. The use of such a model may play a regularization role in their data post-processing whereas, here, the extracted motion fields were subsequently filtered. Their approach was validated over several patients and it was compared with 3D and 4D-CT. Yet, it assumes a standard lung shape, which fails in the diagnosis of patients with differing anatomy like those who underwent lung surgery. They did not explore advanced mechanical characterization with strain, hysteresis, or anisotropy at the voxel size but only basic ventilation parameters (flow and volume) were extracted. Furthermore, mapping remained at the lobar scale with flow-volume loops shown only in the left lung for the upper and the lower lobes. Such spatial resolutions hinder the detection and characterization of small (around 1 cm) and localized lesions or defects in the lung parenchyma.

In any case, an MRI system is far more cumbersome and expensive than a standard spirometer. It is not available today in any pulmonology department and might not be in the near future. Three-dimensional MR Spirometry was performed here with data acquired over 11 min. This total acquisition time may be reduced either by future improvements of the image acquisition procedure or by compromises with the final image quality (voxel size, SNR, under-sampling artifacts, …). Yet, it will be difficult to soon achieve the acquisition of a sufficient amount of data in less than several tens of seconds. This minimal acquisition time covers several quasi-periods of nominal respiratory motion, which requires averaging of data from different respiratory cycles. Even though respiratory motion is not fully repeatable from one motion period to the other, if inspiration and expiration phases are separated and if the outlying motion states are discarded in the post-processing steps as implemented in this work, the motion can fairly be considered repeated for any given MR-gate and time averaged, while spatial resolution can be degraded above the millimeter scale.

The technique we propose demonstrates the advantage to measure directly the mechanical pulmonary behavior in order to estimate local lung function, and the first results shown here are consistent with what is known in pulmonology and to what was observed in previous studies. First, the ventilation feet-head negative gradient, which was initially established by Kaneko *et al*. in 1966 with xenon^133^ ^26^ and recently confirmed by Henderson *et al*. in 2013 with MRI in a single sagittal slice over breath holds^27^, consistently shows up along sagittal and coronal views in both elementary tissue trajectories (Fig. 2) and flow-volume loops (Fig. 7). Second, as assessed by Milic-Emili *et al*. in 1966^28^ and lately reviewed by Clark *et al*. in 2019^29^, the regional volume expansion is enhanced in dependent lung regions. In normal and diaphragmatic breathings, the anterior elementary tissue trajectories (Fig. 2) and Jacobians (Fig. 3) are obviously greater than the posterior ones. Yet, former reference works were based on static measurements during breath holds and dynamic lung regional ventilation still remains controversial. Three-dimensional MR spirometry may provide new insights that will especially clarify the associated position and gravity dependences over the lung function. For example here, in thoracic breathing, the gravity dependence is mitigated by the chest voluntarily-imposed motion so ventilation is made rather uniform in the anterior-posterior direction (Fig. 3). Third, as already observed with 4D Computed Tomography (4D-CT)^30–32^, lung motion hysteresis is clearly depicted by the elementary tissue trajectories (Fig. 2). Finally, the 3D distribution and quantitative values of regional volume expansion and strain anisotropy show strong similarities with recent results obtained in 3D-^33–35^ and 4D-CT^36,37^. ‘Real-time’ 4D-CT would obviously be favored for 3D Spirometry but, contrary to 4D-CT, retrospectively gated MRI is safe for the patient in terms of ionizing radiation, and the number of motion gates is not limited (14 in Jahani *et al*.^36^ and 32 here). Furthermore, the final quality of motion corrected UTE images and the quantity of landmarks observable in the lung with MRI tend to be more and more similar to what can be obtained in motion corrected CT^38,39^.

## Methods

### MR image acquisition protocol

All the acquisitions were performed on a GE Signa PET/MR 3.0 T machine (GE Healthcare, Waukesha, WI, USA). During the imaging process, the subject (male, 26 years old) was lying supine with a respiratory belt around the waist to track his respiratory pattern along the entire imaging process and to eventually verify the plausibility of the self-navigator extracted thereafter. Radiofrequency (RF) excitation was transmitted by the body coil of the PET/MR machine and a 30-channel GE GEM thoracic coil array was used for signal reception. This original study was undertaken according to general clinical research guidelines and regulations. It was approved by our local ethics committee (CEA-SHFJ Scientific Board). Moreover, informed consent was obtained from the volunteer.

A large field of view (FOV) of 320 mm along the superior-inferior and the left-right directions, and 210 mm^22^ along the anterior-posterior direction was chosen with an isotropic voxel size of 1.5 mm. RF excitation was performed with a hard/square RF pulse, without any slice or slab selection gradient, but with phase cycling according to a quadratic law to spoil the remaining transverse magnetization. However, no gradient spoiling strategy was needed thanks to the implementation of an AZTEK trajectory, a k-space trajectory developed in our laboratory, which was adapted to UTE acquisitions to efficiently manage respiratory motion correction with retrospective gating strategies^40^. $TR$ as small as 2 ms was achieved. The flip angle, 3°, was optimized according to the chosen $TR$ and the long ${T}_{1}$ values in the lung parenchyma using Ernst angle formula^41^.

Each spoke (acquired radius in the image k-space) was composed of 192 readout points spread from the center of k-space to the periphery and the readout bandwidth was equal to ±100 kHz. Ramp-sampling was implemented in the pulse sequence to reach a minimal value for $TE$ equal to 12 µs. An oversampling factor around 3 in the number of spokes was found to be a satisfying compromise between the final retrospectively gated image quality – reconstructed with approximately 44,000 spokes and twice more for the reference image at the end of expiration – and the total acquisition time for human studies, which amounted to 10 min and 51 s.

### Motion correction and image reconstruction protocol

The respiratory self-navigator was extracted from the real and imaginary parts of the first point acquired at the beginning of each spoke, for each of the 30 channels of the thoracic reception coil array, thanks to a Principal Component Analysis (PCA). After being checked against the signal outcome from the respiratory belt, this self-navigator was taken as a surrogate signal for respiratory motion correction. This correction was retrospectively applied onto the MR dataset with 32 motion bins defined upon the intrinsic surrogate signal, with 16 bins for the inspiratory phase and 16 bins for the expiratory phase. During the binning process, a view sharing strategy^42^ was performed; outlying respiratory periods were discarded; and spoke weights were computed with a soft-gating approach^43^. A time-stamp was given to each bin. It was computed as the average over the total acquisition duration of the acquisition times of every spoke within the bin, with respect to the time of the end of expiration for the same motion period, taken as the reference motion phase.

The bins were then reconstructed with parallel imaging (iterative SENSE^44^) and compressed sensing^45^ (3D wavelet space L1 minimization) strategies proposed by the open Berkeley Advanced Reconstruction Toolbox (BART^46^). Sensitivity maps for parallel reconstruction were estimated from the k-space data (autocalibration) through the ESPIRiT^47^ method in BART.

### Elastic image registration protocol

Elastic image registration was performed through the open ITK-based Elastix toolbox^48^. In the registration process, the image corresponding to the end of expiration (deflated lung) was taken as the reference (fixed image) and the 31 other images (moving images) were registered onto this reference.

A 3D B-spline transform model characterized by an isotropic control grid size of 20 voxels (30 mm) was chosen for the registration algorithm. A Normalized Mutual Information was chosen as a similarity metric. For each registration process, a multi-resolution strategy^49^ with four smoothing levels was used for both the fixed and the moving images. At each resolution level, 300 iterations of an Adaptive Stochastic Gradient Descent^50^ algorithm were performed with the objective function calculated from 10,000 couples of points located at non-voxel positions and re-sampled randomly at each iteration.

After the 31 image registrations were performed, the extracted deformation fields were temporally smoothed with the ‘smoothn’ algorithm developed on Matlab by the laboratory CREATIS (Lyon, France)^51^.

### Extraction of ventilatory and mechanical features from the deformation fields

After the registration process, 3D deformation fields were obtained along the time-averaged respiratory cycle. The trajectory followed by any elementary piece of lung parenchyma during this average respiratory cycle was graphically represented. From a mathematical modeling point of view, this trajectory is the path defined by the vector $\overrightarrow{r}(X,Y,Z,t)$, which represents the position of the elementary lung volume that was located at position $\overrightarrow{R}=[X,Y,Z]$ at the end of expiration.

From these deformation fields, the deformation gradient tensor $F$ was calculated at each voxel location and at each motion bin time-point as follows:

1 $$F=\left[\begin{array}{ccc}\frac{d{r}_{x}}{dX} & \frac{d{r}_{x}}{dY} & \frac{d{r}_{x}}{dZ}\\ \frac{d{r}_{y}}{dX} & \frac{d{r}_{y}}{dY} & \frac{d{r}_{y}}{dZ}\\ \frac{d{r}_{z}}{dX} & \frac{d{r}_{z}}{dY} & \frac{d{r}_{z}}{dZ}\end{array}\right]$$

The Jacobian of the deformation field was then computed as the determinant of the deformation gradient tensor:

2 $$J={\rm{\det }}(F)$$

It can be demonstrated that $J$ represents the relative volume increase of an infinitesimal sample of tissue centered at the spatial location where $F$ was calculated. If $\delta {V}_{0}$ represents this elementary volume in the reference state, before any deformation, and $\delta V$ the elementary volume after deformation, it can be shown that:

3 $$J=\frac{\delta V}{\delta {V}_{0}}$$

If, at some state, $J > 1$ then the elementary volume has increased with respect to the reference state; if $J < 1$ then it has decreased; and if $J=1$ then it has remained unchanged.

From the knowledge of $F$, several pure strain tensors can be defined and calculated. In this study, the Green-Lagrange strain tensor, written ${\varepsilon }^{G}$, was used:

4 $${\varepsilon }^{G}=\frac{1}{2}({F}^{T}F-I)$$

In the previous expression, $I$ represents the identity matrix. Once diagonalized and expressed in its local orthonormal eigen-basis, ${\varepsilon }^{G}$ can be written:

5 $${\varepsilon }^{G}=[\begin{array}{ccc}{\varepsilon }_{I}^{G} & 0 & 0\\ 0 & {\varepsilon }_{II}^{G} & 0\\ 0 & 0 & {\varepsilon }_{III}^{G}\end{array}]$$

From Eq. (2), it can be demonstrated that $J$ can be well approximated for small strains by the following expressions:

6 $$\begin{array}{ccc}J & \simeq  & 1+{\varepsilon }_{xx}^{G}+{\varepsilon }_{yy}^{G}+{\varepsilon }_{zz}^{G}\\ J\, & \simeq  & 1+{\varepsilon }_{I}^{G}+{\varepsilon }_{II}^{G}+{\varepsilon }_{III}^{G}\end{array}$$

It means that either ${\varepsilon }_{xx}^{G}$, ${\varepsilon }_{yy}^{G}$ and ${\varepsilon }_{zz}^{G}$, or ${\varepsilon }_{I}^{G}$, ${\varepsilon }_{II}^{G}$ and ${\varepsilon }_{III}^{G}$, represent the strain contributions to the regional increase in lung volume respectively along directions $x$, $y$ and $z$ (chosen here to be respectively superior-inferior, left-right and anterior-posterior directions) or along directions $I$, $II$ and $III$ (chosen here such that ${\varepsilon }_{I}^{G} > {\varepsilon }_{II}^{G} > {\varepsilon }_{III}^{G}$). Equation (6) illustrates particularly well the strong relationship between lung regional ventilation and the local mechanical behavior of the organ.

If we consider an elementary sphere of lung parenchyma at the end of expiration, this sphere will take the shape of an ellipsoid oriented along the three eigenvectors found by diagonalizing the Green-Lagrange strain tensor. The ratios between the main radii of the ellipsoid after and before deformation are given by ${\lambda }_{i}=\sqrt{1+2{\varepsilon }_{i}^{G}}$, with $i=I,\,II,\,III$, which are actually the square root of the eigenvalues of ${F}^{T}F$ (see Eq. (4)). Through their relationship with the characteristics of regional deformation fields, ${\lambda }_{I}$, ${\lambda }_{II}$ and ${\lambda }_{III}$ are particularly suited to the calculation of metrics quantifying strain anisotropy. For this study, a Fractional Anisotropy metric, written $FA$, was computed as:

7 $$FA=\sqrt{\frac{{({\lambda }_{I}-{\lambda }_{II})}^{2}+{({\lambda }_{II}-{\lambda }_{III})}^{2}+{({\lambda }_{I}-{\lambda }_{III})}^{2}}{2({{\lambda }_{I}}^{2}+{{\lambda }_{II}}^{2}+{{\lambda }_{III}}^{2})}}$$

This expression is similar to what can be found in the field of diffusion tensor MR imaging to quantify the amount of local anisotropy for the diffusion of water molecules in biological tissues. Here, $FA$ similarly takes values from 0 to 1 and quantifies the amount of strain anisotropy in the lung parenchyma with respect to the end of expiration.

The rate of volume change, $Q$, was derived from $J$ with time by a weighted finite difference method. If ${t}_{n}$ and ${J}_{n}$, $1\le n\le 32$, represent respectively the time-stamp and the Jacobian of the deformation field at one given voxel position, and computed for the n^th^ motion bin, the corresponding regional gas flow ${Q}_{n}$ can be computed as follows:

8 $${Q}_{n}=\frac{1}{{t}_{n+1}-{t}_{n-1}}\left(\frac{{t}_{n+1}-{t}_{n}}{{t}_{n}-{t}_{n-1}}({J}_{n}-{J}_{n-1})+\frac{{t}_{n}-{t}_{n-1}}{{t}_{n+1}-{t}_{n}}({J}_{n+1}-{J}_{n})\right)$$

For $n=1$ and $n=32$, temporal periodic boundary conditions were ensured in order to compute ${Q}_{n}$ for those two extremal bins.

## Supplementary information

Supplementary information.
