# Publisher Correction: 3D Magnetic Resonance Spirometry

**DOI:** 10.1038/s41598-020-72363-2

**Published:** 2020-09-24

**Authors:** Tanguy Boucneau, Brice Fernandez, Peder Larson, Luc Darrasse, Xavier Maître

**Affiliations:** 1 Université Paris-Saclay, CEA, CNRS, Inserm, BioMaps, Orsay, France; 2 Applications & Workflow, GE Healthcare, Orsay, France; 3 Department of Radiology and Biomedical Imaging, University of California San Francisco, San Francisco, CA USA

Correction to: *Scientific Reports* 10.1038/s41598-020-66202-7, published online 15 June 2020

This Article contains an error, where a still image from the animation in the Supplementary Information was inadvertently typeset as Figure 1. The correct Figure 1 appears below.

**Figure 1:**
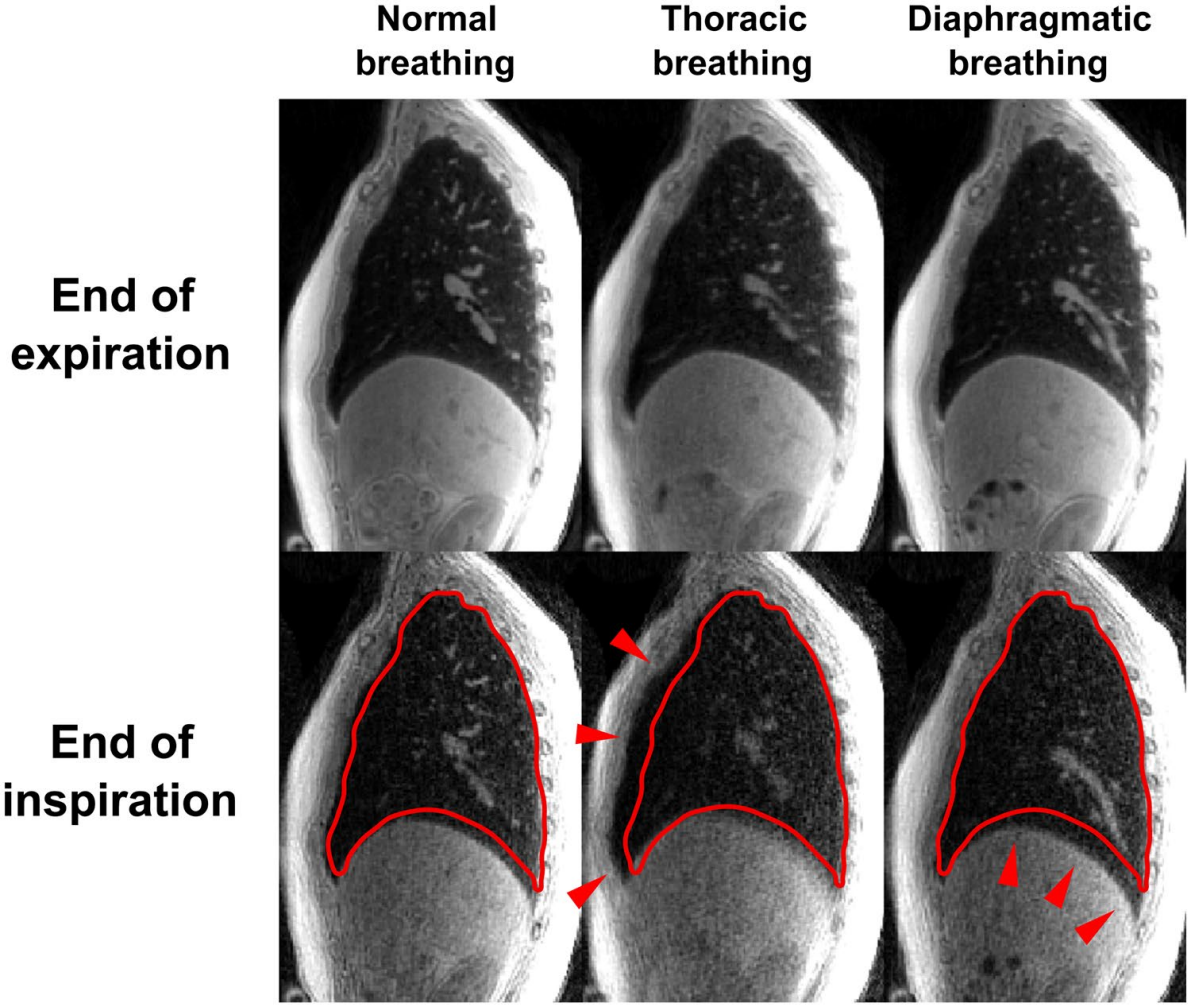
Sagittal views of the thoracic images retrospectively gated at the end of expiration (top) and at the end of inspiration (bottom) for the normal (left), thoracic (middle) and diaphragmatic (right) breathing patterns. On every image at the end of inspiration, the red curve depicts the border of the lung observed in the corresponding expiratory state shown above. As pointed out by the red arrows, it clearly stresses the respective motions of the diaphragm and the thoracic wall for each breathing pattern. The respiratory dynamics are provided as supplementary materials.

