# Supplementary figures and images for: 3D Magnetic Resonance Spirometry
Source: Sci Rep. 2020 Jun 15;10:9649. doi: 10.1038/s41598-020-66202-7 (PMC7295793; doi:10.1038/s41598-020-66202-7)

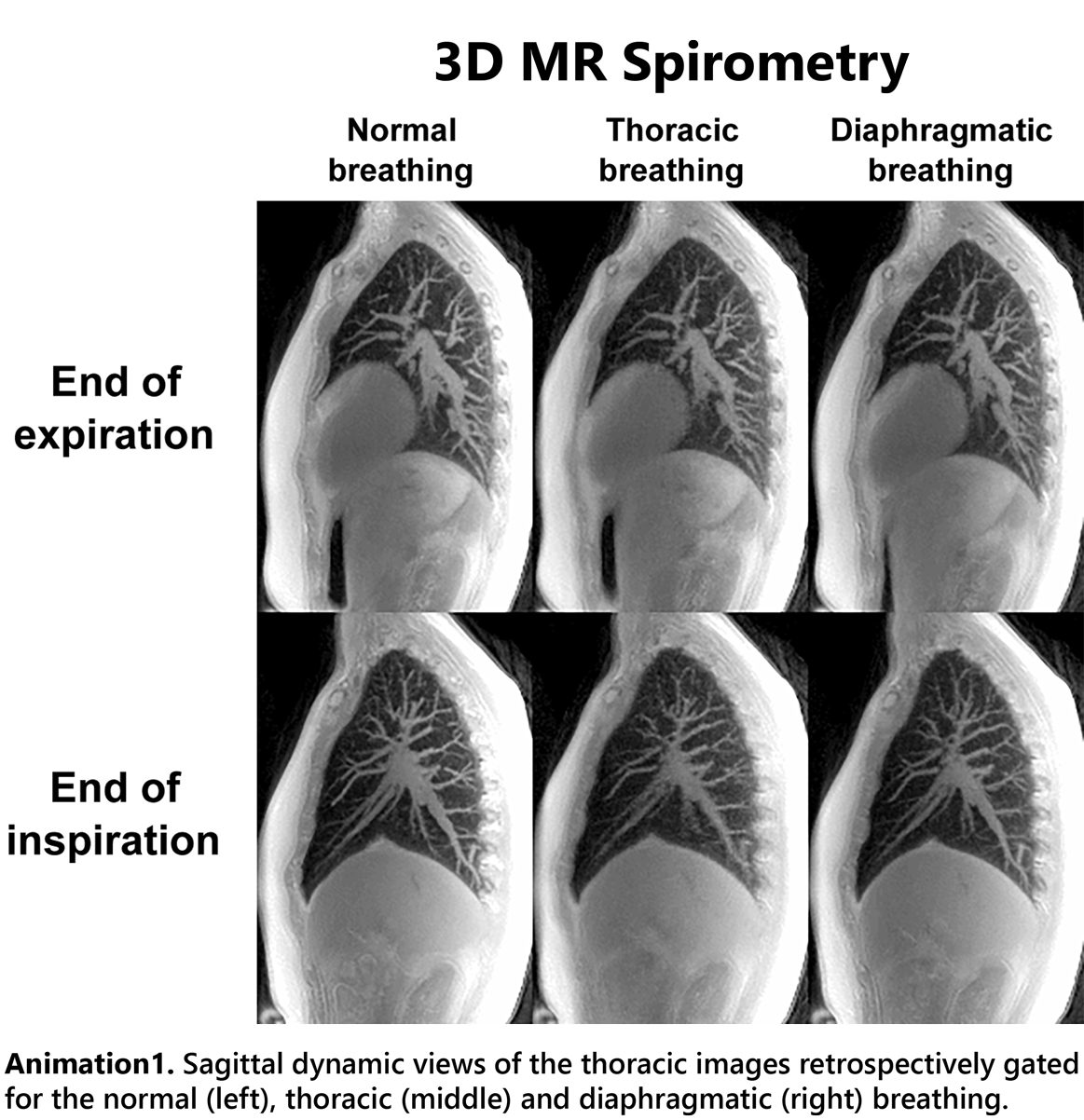

Supplement: Supplementary file 1 — Supplementary information. [file 41598_2020_66202_MOESM1_ESM.gif]
